# *Helicobacter pylori* infection in gastric cancer patients in French Guiana: a hospital-based study (2018–2023)

**DOI:** 10.3389/fgstr.2026.1832175

**Published:** 2026-07-15

**Authors:** Qiannan Wang, Kinan Drak Alsibai, Sarah Bailly, Jean-Pierre Droz, Dominique Louvel, Mathieu Nacher, Alolia Aboikoni

**Affiliations:** 1 Registre des Cancers de Guyane, Centre Hospitalier de Cayenne Andrée Rosemon, Cayenne, French Guiana; 2 Centre d’Investigation Clinique Antilles-Guyane, CIC-INSERM 1424, Centre Hospitalier de Cayenne Andrée Rosemon, Cayenne, French Guiana; 3 Service d’Anatomie et Cytologie Pathologiques, Centre Hospitalier de Cayenne Andrée Rosemon, Cayenne, French Guiana; 4 Université Claude Bernard Lyon 1, Cayenne, France; 5 Service d’Hépato-Gastroentérologie, Centre Hospitalier de Cayenne Andrée Rosemon, Cayenne, French Guiana; 6 Université de la Guyane, Cayenne, French Guiana

**Keywords:** French Guiana, gastric cancer, *Helicobacter pylori*, histology-detected positivity, survival

## Abstract

**Background:**

*Helicobacter pylori* infection is a major etiological factor for gastric cancer, but its prognostic relevance once invasive gastric cancer has developed remains uncertain in French Guiana.

**Methods:**

We conducted a retrospective hospital-based study including all patients diagnosed with histologically confirmed invasive gastric carcinoma at Cayenne Hospital Center between 2018 and 2023. *H. pylori* status was assessed by routine histopathology. Sociodemographic, clinicopathological, and histological characteristics, including associated gastric lesions, were collected. Overall survival was estimated using the Kaplan–Meier method and compared using the log-rank test according to *H. pylori* status and initial management strategy. Exploratory multivariable Cox regression analyses were performed.

**Results:**

Eighty-two patients were included, of whom 43 (52.4%) were *H. pylori*-positive. *H. pylori* positivity was significantly associated with non-active chronic gastritis, whereas *H. pylori* detection was uncommon in active chronic gastritis. *H. pylori* status was not associated with tumor location, histological subtype, differentiation, or preneoplastic lesions. Median follow-up was 25.7 months. Overall survival did not differ between *H. pylori*-positive and *H. pylori*-negative patients. In multivariable analyses, *H. pylori* status was not associated with survival, whereas initial management was strongly associated with outcome, likely reflecting disease stage at diagnosis rather than a causal treatment effect.

**Conclusion:**

Routine histology detected *H. pylori* in about half of gastric tumors, with an association with non-active chronic gastritis. *H. pylori* status was not associated with survival; differences by initial management likely reflected disease severity, with interpretation limited by incomplete TNM staging.

## Introduction

Chronic infection with *Helicobacter pylori* is a major cause of non-cardia gastric cancer and a Group 1 carcinogen, acting through the Correa cascade ranging from chronic gastritis to gastric atrophy, intestinal metaplasia, dysplasia, and invasive gastric carcinoma (1–3). Although gastric cancer incidence has declined in high-income countries, mortality remains high because diagnosis often occurs at advanced stages.

French Guiana, a French overseas territory located in the Amazon region, has a distinctive epidemiological and healthcare context. The population is characterized by marked ethnic and geographic diversity, high levels of social vulnerability, and persistent inequalities in access to healthcare services. Population-based analyses have shown that age-standardized gastric cancer incidence and mortality in French Guiana are higher than mainland France, yet lower than in most South American countries, raising the possibility of setting-specific etiological patterns (4). It has been previously assumed that the local gastric cancer epidemiology reflected a greater prevalence of *H. pylori* infection (4, 5). However, although this is plausible, local data remain limited, and detailed descriptions of tumor characteristics, associated gastric lesions, and risk factors are scarce.

In Latin America, *Helicobacter pylori* infection remains highly prevalent, often exceeding 70% in adult populations, and accounts for a substantial share of regional gastric cancer burden (6, 7). Very high prevalence has been reported in Amazonian and other remote South American territories, frequently attributed to early-life acquisition, household transmission, and limited access to sanitation and healthcare (8, 9). French Guiana shares several structural and environmental determinants with neighboring Amazonian countries, underpinning the hypothesis of high *H. pylori* prevalence among patients with gastric cancer. However, once invasive cancer is established, bacterial detectability may decline, as advanced atrophy and intestinal metaplasia are associated with lower bacterial density and reduced sensitivity of histology-based detection, increasing the risk of false-negative results (10). In parallel, the prognostic value of *H. pylori* infection in gastric cancer remains debated, with inconsistent findings across studies and meta-analyses and potential variation across population and clinical subgroups (11, 12).

We therefore conducted a hospital-based study of patients with gastric cancer managed at Cayenne Hospital Center (2018–2023) to describe tumor histology-detected *H. pylori* positivity, characterize associated clinicopathological features, and explore the relationship between *H. pylori* status and overall survival.

## Methods

### Study design and target population

This retrospective observational hospital-based study was conducted at Cayenne Hospital Center. All patients with a histologically confirmed diagnosis of invasive gastric carcinoma (ICD-O-3 C16, morphology/3) between 2018 and 2023 were included, based on pathology reports. Gastric lymphomas and *in situ* carcinoma were excluded due to their distinct nosological entities and prognostic profiles.

### Data collection

Collected data included sociodemographic characteristics (date of diagnosis, date of birth, sex, place of birth), tumor characteristics (gastric location, histological type and subtype, and degree of differentiation), and associated gastric lesions, including chronic gastritis activity categorized as active or non-active according to pathology reports, as well as atrophy, intestinal metaplasia, and dysplasia. *Helicobacter pylori* status was determined retrospectively from routine histopathology reports available at diagnosis and was therefore analyzed as histology-detected H. pylori positivity rather than as true infection prevalence or lifetime exposure. No systematic multimodal or gold-standard diagnostic strategy was used for case classification. Rapid urease testing, serology, urea breath testing, stool antigen testing, and PCR were not systematically available. The specific staining method, exact sampling site, and number of gastric sites sampled were not consistently specified in the pathology reports.

Information regarding initial management was extracted from medical records and categorized into treatment groups: surgery plus chemotherapy, surgery alone, chemotherapy alone, palliative care, or undocumented management. Follow-up was conducted until December 31, 2025.

### Statistical analysis

Histology-detected *H. pylori* positivity was described overall and stratified by sex, place of birth, and selected tumor and histological characteristics.

Patient characteristics were described overall and according to *H. pylori* status. Comparisons between *H. pylori*-positive and *H. pylori*-negative groups were performed using the Wilcoxon rank-sum test for quantitative variables. For qualitative variables, Pearson’s chi-square test with Yates’ continuity correction was used when expected cell counts were sufficient; Fisher’s exact test was applied when expected counts were small.

Median follow-up time was estimated using the reverse Kaplan–Meier method, treating censoring as the event of interest. Overall survival was estimated using the Kaplan–Meier method and survival curves were compared using the log-rank test, according to *H. pylori* infection status and initial management category. For survival analyses by treatment, patients with undocumented initial management were excluded.

Overall survival was further analyzed using Cox proportional hazards regression. The primary multivariable model included *H. pylori* status, age at diagnosis, and documented initial management, selected *a priori* based on clinical relevance. Results are presented as hazard ratios (HRs) with 95% confidence intervals (95% CI). All statistical significance was defined as of p < 0.05. As sensitivity analyses, the model was additionally adjusted for sex and migration status. To further assess the stability of variable selection in the context of a limited number of events, penalized Cox regression using LASSO and elastic-net penalties with cross-validation was performed among prespecified candidate variables. All statistical analyses were performed using R software (version 4.4.2).

### Ethical and regulatory considerations

The study was conducted in accordance with the General Data Protection Regulation (GDPR) and the French regulatory framework. Data processing was authorized by the French Data Protection Authority (CNIL; authorization number DT2230950). Patients were informed of the use of their data and could object in accordance with procedures applicable to cancer registry.

## Results

### Histology-detected *H. pylori* positivity among gastric cancer patients

Histology-detected *H. pylori* positivity among patients with gastric cancer is summarized in **Table 1**. Overall, *H. pylori* was identified in 43 of 82 patients (52.4%). Histology-detected positivity was slightly higher in women than men (58.1% vs. 49.0%) and varied markedly by place of birth, from 33.3% among patients born in Haiti to 77.8% among those born in Suriname; positivity was 50.0% in patients born in French Guiana and 55.6% in those born in Brazil.

**Table 1:** Histology-detected *H. pylori* positivity according to patient characteristics.

Characteristic	Category	*H. pylori*-positive / total	Histology-detected *H. pylori* positivity (%)
Total	All patients	43 / 82	52.4
Sex	Female	18 / 31	58.1
	Male	25 / 51	49.0
Place of birth	French Guiana	13 / 26	50.0
	Brazil	10 / 18	55.6
	Haiti	5 / 15	33.3
	Suriname	7 / 9	77.8
	Others	8 / 14	57.1
Tumor location	Pyloric	3/12	25.0
	Non-Pyloric	40/70	57.1
Histological subtypes	Tubular (intestinal type)	31 / 62	50.0
	Signet-ring cell (diffuse type)	8 / 13	61.5
	Other (mixed/rare)	4 / 7	57.1
Associated gastric lesions	Chronic gastritis (active)	3 / 18	16.7
	Chronic gastritis (non-active)	40 / 64	62.5
	Atrophy (present)	29 / 53	54.7
	Intestinal metaplasia (present)	28 / 52	53.8
	Dysplasia (present)	14 / 22	63.6

*H. pylori* positivity also differed across pathological features. It was 50.0% in tubular carcinomas and 61.5% in signet-ring cell carcinomas. Detection was uncommon in the presence of active chronic gastritis (16.7%) but frequent in the presence of non-active chronic gastritis (62.5%). Higher positivity was also observed among patients with advanced preneoplastic lesions, particularly dysplasia.

### Patients characteristics by *H. pylori* status

Baseline clinicopathological characteristics according to *H. pylori* status are shown in **Table 2**. A total of 82 patients with invasive gastric carcinoma were included, 43 were *H. pylori*–positive.

**Table 2:** Patient characteristics according to Helicobacter pylori infection status.

Characteristics	Total (n=82)	*H. pylori*-negative (n=39)	*H. pylori*-positive (n=43)	*p-value*
Demographic characteristics				
Age at diagnosis, median (IQR), years	67.1 (54.6–73.1)	69.6 (55.3–73.4)	64.6 (53.7–72.9)	0.64^a^
Sex, n (%)				0.57^b^
— Female	31 (37.8)	13 (33.3)	18 (41.9)	
— Male	51 (62.2)	26 (66.7)	25 (58.1)	
Place of birth, n (%)				0.33^c^
— French Guiana	26 (31.7)	13 (33.3)	13 (30.2)	
— Brazil	18 (22.0)	8 (20.5)	10 (23.3)	
— Haiti	15 (18.3)	10 (25.6)	5 (11.6)	
— Suriname	9 (11.0)	2 (5.1)	7 (16.3)	
— Others	14 (17.0)	6 (15.4)	8 (18.6)	
Tumors characteristics				
Tumor location, n (%)				0.059^c^
— Pyloric	12 (14.6)	9 (23.1)	3 (7.0)	
— Non-pyloric	70 (84.4)	30 (76.9)	40 (93.0)	
Histological subtype, n (%)				0.75^c^
— Tubular	62 (75.6)	31 (79.5)	31 (72.1)	
— Signet-ring cell	13 (15.9)	5 (12.8)	8 (18.6)	
— Other (mixed/rare)	7 (8.5)	3 (7.7)	4 (9.3)	
Differentiation, n (%)				0.78^b^
— Well/moderately differentiated	46 (56.1)	23 (59.0)	23 (53.5)	
— Poorly/undifferentiated	36 (43.9)	16 (41.0)	20 (46.5)	
Associated gastric lesions (pathology)				
Chronic gastritis type, n (%)				0.0015^c^*
— Non-active chronic gastritis	64 (78.0)	24 (61.5)	40 (93.0)	
— Active chronic gastritis	18 (22.0)	15 (38.5)	3 (7.0)	
Atrophy (present), n (%)	53 (64.6)	24 (61.5)	29 (67.4)	0.74^b^
Intestinal metaplasia (present), n (%)	52 (63.4)	24 (61.5)	28 (65.1)	0.92^b^
Dysplasia (present), n (%)	22 (26.8)	8 (20.5)	14 (32.6)	0.33^b^

^a^Wilcoxon rank-sum test.

^b^Pearson’s χ² test with Yates’ continuity correction.

^c^Fisher’s exact test.

*p < 0.05.

The median age at diagnosis was 67.1 years (IQR 54.6–73.1). Patients with *H. pylori* – positive were slightly younger age than those in the *H. pylori* – negative group (64.6 vs. 69.6 years), but this difference was not significant (p=0.64). Men accounted for 62.2% of the cohort, with a similar sex distribution in both groups. Among H. pylori-positive patients, 30.2% patients were born in French Guiana and 23.3% in Brazil, with the remaining patients distributed across several countries of birth (p=0.33).

Most tumors were located outside the pylorus (85.4%). Non-pyloric location tended to be more frequent among *H. pylori*–positive patients, although the difference did not reach statistical significance (93.0% vs 76.9%; p = 0.059). Tubular carcinoma was the predominant histological subtype (75.6%), followed by signet-ring cell carcinoma and other subtypes; subtype distribution and tumor differentiation were comparable between *H. pylori* – positive and – negative patients.

Chronic gastritis was observed in 78.0% of patients. Non-active chronic gastritis was significantly more frequent among *H. pylori*–positive patients than among *H. pylori* – negative patients (93.0% vs. 61.5%), while active chronic gastritis was more common in *H. pylori* – negative patients (38.5% vs. 7.0%; p = 0.0015). By contrast, the presence of atrophy, intestinal metaplasia, and dysplasia did not differ significantly according to *H. pylori* infection status.

### Overall survival

**Figure 1:**
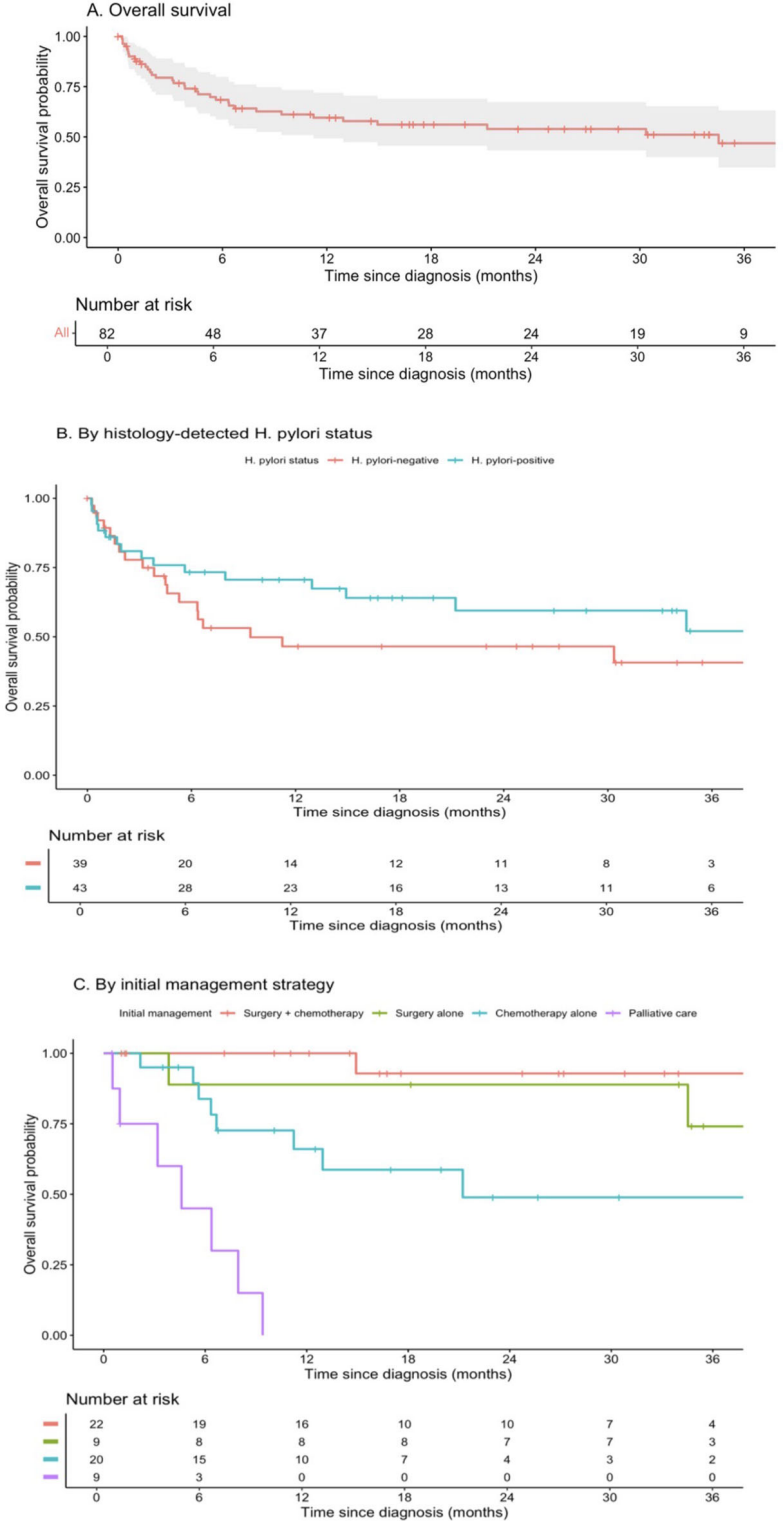
Kaplan-Meier overall survival curves among patients with gastric cancer managed at Cayenne Hospital Center (2018-2023). **(A)** Overall survival in the full cohort. **(B)** Overall survival according to histology-detected *H. pylori* status. **(C)** Overall survival according to initial management strategy. Risk tables indicate the number of patients at risk over time.

During follow-up, 38 deaths occurred and 44 patients were censored, including 17 patients lost to follow-up. Median follow-up time, estimated using the reverse Kaplan–Meier method, was 25.7 months (95% CI, 17.6–34.0). Median overall survival was 34.5 months (95%CI, 11.2 months–not reached). The 12-, 24- and 36-month overall survival probabilities were 59.6% (95%CI, 49.3–72.0), 54% (95% CI, 43.3–67.3), and 46.9% (95% CI, 34.8-63.2), respectively. The 36-month estimate should be interpreted with caution because only nine patients remained at risk at that time point. The Kaplan–Meier curve showed an early and pronounced decline in survival, with the steepest drop occurring within the first 6 months after diagnosis (**Figure 1A**).

Overall survival did not differ significantly by *Helicobacter pylori* infection status (log-rank p = 0.39), and survival curves for *H. pylori* – positive and – negative patients were largely superimposed throughout follow-up (**Figure 1B**), although survival estimates tended to be slightly higher among *H. pylori* – positive patients.

By contrast, overall survival varied substantially according to initial management strategy (log-rank p < 0.001) (**Figure 1C**). Survival was highest among patients initially managed with surgery plus chemotherapy. Patients receiving chemotherapy alone showed lower survival, with a gradual decline over time. Patients managed with palliative care had the poorest outcomes, with a sharp drop in survival within the first months after diagnosis.

### Multivariable survival analysis

The exploratory Cox regression model included 60 patients with documented initial management, among whom 21 deaths occurred. After adjustment for age at diagnosis, histology-detected *H. pylori* status, and initial management, *H. pylori* positivity was not associated with overall survival. Survival differed across initial management categories, with higher hazards observed among patients treated with chemotherapy alone or managed with palliative care compared with those receiving surgery plus chemotherapy (**Table 3**). However, these estimates should be interpreted cautiously because of the limited sample size, small number of events, and wide confidence intervals. Initial management should be interpreted as a marker of disease extent, resectability, performance status, comorbidities, access to care, and eligibility for curative-intent treatment, rather than as a causal determinant of survival.

**Table 3:** Exploratory multivariable Cox regression analysis for overall survival.

Variable	HR	95 % CI	*p-value*
Age at diagnosis	0.99	0.96 – 1.02	0.38
*Helicobacter pylori* infection status			
H. pylori-negative	Reference	—	—
H. pylori-positive	0.78	0.30 – 2.01	0.60
Initial treatment			
Surgery + chemotherapy	Reference	—	—
Surgery alone	2.65	0.34 – 20.93	0.35
Chemotherapy alone	7.89	1.67 – 37.23	0.009*
Palliative care	56.89	9.82 – 329.45	< 0.001*

*p < 0.05.

In sensitivity analyses, penalized Cox regression using LASSO and elastic net penalties with cross-validation retained initial management as the main selected variable under the least conservative penalty. Given the limited sample size and number of events, these analyses were considered exploratory and used only to assess the stability of variable selection.

## Discussion

In this hospital-based cohort of invasive gastric cancer in French Guiana, *Helicobacter pylori* was identified in about half of tumors by routine histology. Overall survival did not differ by *H. pylori* status, whereas initial treatment strategy was associated with differences in prognosis.

The association between histology-detected *H. pylori* positivity and non-active chronic gastritis may be compatible with a late-stage “burnt-out” pattern. As lesions progress along the Correa cascade, bacterial detectability may decline, and routine histology on tumor-associated mucosa can yield false-negative results when organisms are sparse, sampling is limited, or advanced mucosal changes such as atrophy and intestinal metaplasia are present. Previous eradication therapy, recent antibiotic exposure, or proton pump inhibitor use may further reduce histological detection, although these data were not consistently available in our retrospective cohort. Conversely, PCR may detect low-density infection (or residual DNA), increasing apparent prevalence compared with histology (10).

In mainland France, *H. pylori* prevalence in the general adult population is commonly reported around 15-30% (13); in French Guiana, prevalence is likely higher. A Cayenne Hospital Center study using systematic PCR on gastric biopsies obtained during routine endoscopy showed an *H. pylori* prevalence of approximately 45%, with women representing 53.7% of infected patients (14). This finding contrasts with the consistent male predominance in gastric cancer incidence worldwide (15) and may suggest that sex differences in progression from infection to cancer may exist, potentially involving hormonal influences, immune responses, attitudes towards health care, and lifestyle exposures (16–19).

Recent IARC regional evidence from Latin America and the Caribbean indicates that *H. pylori*-related gastric cancer remains the predominant pattern in the region, with late diagnosis contributing to poor outcomes and substantial variation in *H. pylori* prevalence across countries (20). A recent hospital-based study comparing gastric cancer cohorts from Europe and Latin America reported differences in clinicopathological profiles and treatment pathways, with poorer overall survival in the Latin American cohort (21). Comparison with South American cohorts underscores wide variability in tumor *H. pylori* detection, largely related to diagnostic strategy and sampling. A Peruvian quantitative PCR-based series reported a prevalence of 60.8% prevalence among 375 gastric cancers (22). In a Brazilian gastrectomy series using combined histologic and microbiologic methods with multiple non-atrophic sampling sites, *H. pylori* was detected in 82.5% of cases (n=40), and highlighted that combining methods improves detection when bacterial density is low in carcinoma tissue (23). In our cohort, routine histology identified *H. pylori* in 52.4% of tumors (43/82). When case counts were compared, the difference versus Peru was not statistically significant (χ², p=0.16), whereas the difference versus Brazil was statistically significant (χ², p=0.001). These comparisons should be interpreted cautiously given the higher sensitivity of PCR and multimodal approaches; nevertheless, the pattern supports diagnostic heterogeneity and likely under-detection by histology, particularly in advanced lesions.

Evidence on prognosis is heterogeneous, with some studies reporting better survival among *H. pylori*-positive gastric cancer patients (24–27), while others found no clear association (28). In our cohort, histology-detected *H. pylori* positivity was not associated with overall survival. However, tumor stage was largely unavailable and could not be included in the survival analyses. Because stage is closely linked to *H. pylori* detectability, treatment selection, and survival, residual confounding by disease severity cannot be excluded. Initial management should therefore be interpreted as a marker of disease extent, resectability, performance status, and eligibility for curative-intent care, rather than as a causal treatment effect.

Given the marked population heterogeneity in French Guiana, variation in *H. pylori* strains may also contribute to gastric carcinogenesis. Virulence factors, particularly CagA and VacA, are known to influence the carcinogenic potential of *H. pylori* (29, 30). However, genotypic characterization of H. pylori strains was not available in our cohort, preventing assessment of whether regional strain variation contributed to the observed clinicopathological patterns or survival outcomes. Place-of-birth differences in tumor *H. pylori* detection were observed, but small strata and histology-based ascertainment limit inference; these findings should be considered exploratory. Future studies incorporating bacterial genotyping, dietary exposures, and household transmission patterns could help better characterize gastric carcinogenesis in French Guiana.

This study is limited by its retrospective single-center design, modest sample size, loss to follow-up, largely incomplete TNM staging, and reliance on routine histopathology for *H. pylori* assessment. Key prognostic variables, including performance status, comorbidities, longitudinal treatment details, nutritional and inflammatory markers, and dietary exposures, were not consistently documented, preventing adjustment for composite prognostic indices such as the Cachexia Index (31). In addition, the study period overlapped with the COVID-19 pandemic, which may have affected diagnostic pathways, treatment initiation, tumor stage at presentation, and baseline nutritional status; however, these effects could not be formally assessed in our dataset (32).

Within these limits, *H. pylori* status at diagnosis does not appear to be a major determinant of prognosis in this setting. Survival differences according to initial treatment pathway most likely reflected disease extent, resectability, performance status, and eligibility for curative-intent care, rather than a causal effect of treatment category itself. Priorities for improving outcomes include earlier diagnosis and timely access to curative-intent management. From a prevention perspective, these findings support upstream strategies focused on earlier detection and eradication of *H. pylori* before advanced mucosal changes occur, while further research is needed to identify populations in whom such interventions would be most effective.

## Conclusion

In this hospital-based cohort of patients with invasive gastric cancer in French Guiana, routine histology detected *H. pylori* in about half of gastric tumors. This proportion, higher than that typically reported in mainland France but lower than in some Brazilian series, may reflect French Guiana’s intermediate epidemiological position between Europe and the Amazon region. Histology-detected *H. pylori* positivity was associated with non-active chronic gastritis, a pattern that may reflect reduced bacterial detectability late in carcinogenesis. *H. pylori* status at diagnosis was not associated with overall survival, while survival differences according to initial management likely reflected disease severity and eligibility for curative-intent care rather than a causal treatment effect.

## Data Availability

The datasets presented in this article are not readily available because they contain patient-level clinical information and are subject to privacy and data protection regulations. Data were collected from hospital medical records and are therefore subject to institutional and regulatory restrictions. De-identified data may be made available from the corresponding author upon reasonable request and with appropriate institutional approvals. Requests to access the datasets should be directed to qiannan.wang@ch-cayenne.fr.
